# Teacher identification of reading difficulties among Arabic-speaking third graders in Israel: a pilot study

**DOI:** 10.1007/s11881-025-00331-4

**Published:** 2025-06-07

**Authors:** Sumod Khatib-Abbas, Orly Lipka

**Affiliations:** 1 Department of Learning Disabilities, University of Haifa, Haifa, Israel; 2 Edmond J. Safra Brain Research Centre for the Study of Learning Disabilities, Department of Learning Disabilities, Faculty of Education, University of Haifa, Haifa, Israel

**Keywords:** Arabic-speaking teachers, Decision-making, Identification, Reading comprehension, Reading fluency, Students at risk of reading difficulties

## Abstract

Recently, concerns have been raised about Arabic-speaking students’ reading achievement in Israel. Understanding language teachers’ ability to identify poor reading skills is crucial to improve students’ literacy outcomes. This pilot study examined three main aspects: the factors Arabic-speaking language teachers use to determine their third-grade students’ word reading fluency and comprehension, their ability to identify students who need an intervention based on their reading performance, and the differences between teachers who make accurate and those who make inaccurate assessments of their students’ literacy skills. The pilot study included a preliminary sample of 58 teachers and 112 students, with one to three students selected from each teacher’s classroom for assessment. All participants were native Arabic speakers. Initial findings suggest that the main factors informing teachers’ decisions on reading fluency and comprehension are vocabulary size (65.70%–77.70%), linguistic skills (63.9%–76%), and oral reading level (62%–74.30%), while less emphasis was placed on test scores (25.9%–31%) and parents’ reports (13.9%–32.80%). Four distinct profiles of students at risk of reading difficulties (ARORD) emerged: low word reading fluency and comprehension (22%), low fluency only (12%), low comprehension only (4%), and a typical group (62%). Teachers identified students with difficulties in both areas with 80% accuracy, in reading comprehension with 60%, in word reading fluency with 0%, and in the typical group with 65%. The data indicated that teachers who taught more student-facing hours were better at identifying students ARORD. The implucations highlighte the need for teachers training focused on enhancing Arabic teachers' ability to accurately assess literacy skills and become familiar with different profiles of students’ reading difficulties.

## Introduction

The unique linguistic characteristics of Arabic pose challenges for teachers and students in the teaching and acquisition of literacy skills. Students who speak Arabic face difficulties in acquiring these skills, primarily due to the distinct features of the Arabic language (Levin et al., 2008). Previous researchers (Asaad & Eviatar, 2014; Saiegh-Haddad, 2007) identified three main characteristics that raise challenges for Arabic readers: (a) diglossia; (b) the visual characteristics of Arabic orthography; and (c) the complex relationship between graphemes and phonemes. These factors collectively contribute to the complexities of Arabic literacy acquisition. An additional source of difficulty in Arabic reading acquisition is the presence of many visual and phonological neighbors, which are letters that share a basic form and differ only in placement and number of dots (Asaad & Eviatar, 2014). Arabic reading acquisition, as in many languages, extends well beyond the elementary years due to the language’s complex orthography, diglossia, lack of vowelization in most texts, and rich morphology. This aligns with Chall’s (1983) lifelong reading development model. The orthographic challenges and vowel decoding when learning to read Arabic necessitate advanced cognitive functions, requiring that readers balance resources between decoding and comprehension (Asadi & Ibrahim, 2012). This supports claims that Arabic reading proficiency continues to develop long after the initial years of instruction.

Israel’s public education system has a complex hierarchical structure, reflecting the country’s socio-cultural diversity. It is divided into two main sectors: the Jewish and the Arab education systems. Each operates as an independent unit with unique characteristics—from the student population through to the teaching staff and curriculum (Abu-Saad & Khalil, 2009). This structural division is particularly evident in language education policies and practices. Arabic serves as the national language and first language for the large Arabic minority in Israel (Suleiman, 2018). Consequently, students in Arabic-speaking schools begin their education in Modern Standard Arabic (MSA) as their first language, which differs significantly from their spoken Arabic dialects (Poyas & Bawardi, 2018). However, Hebrew holds the position of the main and official language in Israel, which is extensively employed by state institutions and dominates the public sphere (Kawar, 2024). Due to the importance of Hebrew acquisition, it is a mandatory subject in Arabic-speaking schools. In most Arab sectors, formal instruction typically begins in the third grade with four to five hours per week, except for Druze Arabs, for whom it starts earlier, with two hours per week in second grade, increasing to five hours from third grade (Ministry of Education, 2017). Moreover, in addition to Arabic and Hebrew, Arab students begin studying English from the third grade (Manor & Binhas, 2023). As such, these students face the challenging task of acquiring two languages simultaneously alongside their mother tongue, which requires that they navigate a complex trilingual educational environment.

Saiegh-Hadad (2018) proposed the MAWRID model of Arabic word reading, where its development is shaped by three main features: (a) diglossia, (b) the root-and-pattern morphology, and (c) orthographic complexity. Diglossia was defined by Ferguson (1959) as a relatively stable linguistic situation where two forms of a language are used in the same community. In the Arabic-speaking Israeli community, the first of these languages is spoken Arabic and the second is standard Arabic, which is usually more grammatically complex and is mostly used in formal written contexts (Ferguson, 1959). This standard Arabic is studied formally within the education system, while spoken Arabic is acquired at home and used in daily life (Poyas & Bawardi, 2018).

The root-and-pattern morphological structure of the Arabic language plays an important role in reading acquisition (Abu-Rabia, 2007; Saiegh-Haddad & Geva, 2008). In Semitic languages, most content words are made up of at least two abstract morphemes: a root and a word pattern. The root in Arabic is typically triliteral, composed of three consonants representing the basic semantic meaning (Saiegh-Haddad & Henkin-Roitfarb, 2014). For example, the Arabic root (ك-ت-ب) (k-t-b) has the basic meaning “writing,” which can be combined with different word patterns to derive related such as كَتَبَ (kataba)—“he wrote” and كِتَاب (kitāb)—“book.” This root-and-pattern structure, which is fundamental to Semitic languages, enables rich morphological complexity. Roots can also have two or four consonants, always indicating the word’s core meaning. Word patterns are phonological, including the vowels intercalated among the root consonants (Saiegh-Haddad & Henkin-Roitfarb, 2014).

The Arabic alphabet consists of 29 letters, of which 6 are vowels, and the Arabic language also includes diacritics (e.g., quiescence [sukūn, ْ], a double letter stress [shaddah, ّ], and nunation [tanwin, ً ٌ ٍ]; Poyas & Bawardi, 2018; Saiegh-Haddad, 2018). The Arabic writing system has two script versions: deep and shallow orthography. Voweled orthography is considered shallow orthography because it gives the reader full phonological information about the written word (e.g., كَتَبَ [kataba]—“he wrote”). In contrast, the unvoweled orthography is considered deep orthography since it is written without any diacritical marks (e.g., كتب [ktb]), resulting in a lack of phonological information, which often leads to reading difficulties (Abu-Rabia & Taha, 2004; Poyas & Bawardi, 2018; Saiegh-Haddad, 2018). With respect to graphemes and phonemes, of the Arabic language’s 29 letters, 23 have different shapes according to their position in the word—initial, middle, final, or separate (e.g., the letter “ع” [ayn] can appear as “عـ” at the beginning, “ـعـ” in the middle, “ـع” at the end, or “ع” when separate), while the other six letters have two different shapes—final or separate (e.g., the letter “و” [waw] appears as “ـو” at the end of a word or “و” when separate; Asaad & Eviatar, 2014).

In Israel’s Arabic-language education system, kindergarten and first grade are focused on learning and recognizing letters’ forms and remembering their names and sounds. Here, students must be taught to connect the shapes of letters with their corresponding sounds (e.g., “ب” [bā’] represents the sound “b”; Shibel, 2008). First- and second graders start learning to read in MSA (Asaad & Eviatar, 2014), while in third grade, the focus moves away from basic reading abilities, and students are required to use reading in order to learn other topics. This transition alters the contributions of linguistic and cognitive components to reading (Asadi & Ibrahim, 2012). Then, midway through third grade comes the transition from voweled to unvoweled text (e.g., from كَتَبَ [kataba] to كتب [ktb], both meaning “he wrote”). Here, third-grade students transition from analytical reading (reading each letter and its vowels separately) to global reading (recognizing whole words at once), which enables students to understand a text’s general meaning. However, they may struggle to read unvoweled Arabic text fluently because of written Arabic’s visual complexity (e.g., distinguishing between similar-looking letters such as “ب” [bā’], “ت” [tā’], and “ث” [thā’]; Asaad & Eviatar, 2014).

The results of the PIRLS 2021 computerized test pilot study highlight concerning trends in Israel’s reading achievement. In the study, Arabic-speaking students comprised 25% of the total participants, while Hebrew-speaking students made up 75%, reflecting the demographic composition of Israel’s student population. While Israel’s average score was 510 points, slightly above the international average of 501, a critical situation was revealed for Israel’s Arabic-speaking students, with 57% of fourth-grade Arabic speakers struggling to read, which is approximately double the international average. Moreover, the average score in Israel for Arabic-speaking students was 454, far lower than the 529 for Hebrew speakers. Furthermore, the achievement gap between Hebrew and Arabic speakers was found to have narrowed, but this was not due to improvements among Arabic speakers. Instead, it was the result of Hebrew speakers’ significant decline in performance. These findings underscore the challenges in achieving reading proficiency across both language groups under Israel’s education system, with Arabic-speaking students representing a significant minority facing particular difficulties.

In the 2022/2023 academic year, Israel’s national Meitzav tests were taken in Arabic by fourth- and eighth-grade students, assessing their reading comprehension based on that expected of them in the Arabic-language curriculum, and the results showed that 88% and 75% of Arabic-speaking students in the fourth and eighth grades, respectively, struggled with reading comprehension in their mother tongue (RAMA, 2023).

Studies indicate that many teachers lack deep knowledge in fundamental areas of language, such as structure and morphology. Additionally, they lack the phonological awareness and research-based teaching methods required for language development (Hammond, 2015; Spear-Swerling & Zibulsky, 2014). Accordingly, they can struggle to effectively support early readers and identify those requiring an intervention in a timely manner. The Arabic language poses unique challenges in this context, both for teachers and students. A significant obstacle is teachers’ lack of knowledge and tools for identifying students at risk of reading difficulties (ARORD) in the third grade, a critical period for reading development. In this context, teachers’ decision-making plays a crucial role in identifying at-risk students and determining their eligibility for special education support (Begeny et al., 2011; Martin, 2005). Therefore, it is essential that we examine teachers’ decision-making processes, to identify opportunities for enhancing their ability to make informed choices and thus supporting the earlier identification of at-risk students and the timelier implementation of appropriate interventions.

## Teachers’ decision-making regarding students’ literacy skills

Teachers’ decision-making is an important evaluative tool in the educational framework for students ARORD (Martin, 2005), which dictates whether and when these students are identified. During instruction, teachers are required to make decisions regarding students’ academic performance (Clark & Peterson, 1986; Martin, 2005; Schmitterer & Brod, 2021; Seastrand, 2022). In many educational environments, teachers freely choose which information or methodology they use to identify students ARORD (Schildkamp & Kuiper, 2010). Teachers and school leaders can use student performance data, for instance, to justify changes to the teaching methods in their schools and to increase student achievement (Lai & Schildkamp, 2013). In this way, teachers’ decision-making may directly affect students’ achievement (Cadwell & Jenkins, 1986), with far-reaching implications for teaching practice and students’ future academic pathways (Begeny et al., 2011; Urhahne & Wijnia, 2021).

Education systems use different methods to identify students who need additional support and interventions. For instance, teachers may use information from standardized tests in order to monitor students’ individual development and provide additional support to students with learning needs (for the Response-to-Intervention approach, see Compton et al., 2006). However, there has been little research on the information teachers with informal approaches use to reach their decisions.

Teachers’ decision-making should be based on a range of systematic information (Lai & Schildkamp, 2013). Ikemoto and Marsh (2007) distinguished different types of data teachers may use in their decision-making, such as cognitive outcome data (i.e., student performance), input data (i.e., student background), process data (i.e., quality of instruction in the classroom), and satisfaction data (i.e., opinions of educational staff, students, and their parents; Ikemoto & Marsh, 2007). Schmitterer and Brod (2021) examined which kinds of factors teachers used when they assessed their students, finding that teachers relied mostly on outcome data, including reading abilities and linguistic abilities connected to reading fluency, and to a lesser extent, school grades, parents’ reports, and self-motivation. Meanwhile, Ingram et al. (2004) found that teachers tended to rely on intuition and experience rather than systematic data.

## Accuracy of teachers’ decision-making

Teachers’ judgment accuracy refers to the precision of their knowledge about various student characteristics, including cognitive abilities, learning motivation, and social behaviors (Urhahne & Wijnia, 2021). This has importance as it may directly impact instructional choices. As Shavelson (1978) noted, these judgments “provide essential information for deciding what and how to teach” (p. 37). Furthermore, Urhahne and Wijnia (2021) emphasize the crucial role of accurate teacher judgments in effective teaching. Specifically, they assert that teachers who can accurately assess individual differences among students can continually adapt their teaching methods. Consequently, this adaptability allows teachers to modify their social interactions, communication styles, and instructional approaches to better suit each student’s needs. In essence, the better teachers can gauge their students’ mental states and learning needs, the better equipped they are to tailor their teaching methods and make decisions that positively impact student learning.

Hoge and Coladarci (1989) reviewed 16 studies published between 1962 and 198, finding that all but one examined the reading domain, and that only four examined reading comprehension. The reviewers examined the match between (a) teachers’ decision-making regarding their students’ academic abilities and (b) the students’ actual academic performance, finding a high positive correlation between the two (*r* = 0.66).

Different measurement tools can assess and identify students ARORD. Previous studies of teachers’ accuracy in this identification relied mainly on norm-referenced measures for evaluating students’ achievement in reading and mathematics (Eckert et al., 2006). Recently, researchers have used curriculum-based measurement (CBM) procedures to measure students’ performance (Eckert et al., 2006; Feinberg & Shapiro, 2003). CBM is a classroom method that teachers may use to determine students’ academic levels (Mukherjee, 2008). Madeline and Wheldall (2005) examined teachers’ decision-making accuracy when using a reading fluency measure compared to a curriculum-based measure of students’ reading performance in the third and fifth grades, finding that only 50% of teachers identified the poorest reader.

Schmitterer and Brod (2021) examined how teachers decided whether third-grade students should attend a remedial reading program. The students participated in individual and group sessions where they completed several tasks (on word reading, sentence reading, text reading, spelling, phonological awareness, and vocabulary size). Teachers identified on average almost 50% of their students as “definitely” or “possibly” needing a reading intervention, while 25% to 40% of those students achieved normal scores on the standardized tests. Urhahne and Wijnia (2021) also reported on the lack of absolute accuracy in teacher judgments. Their analysis revealed a concerning trend—teachers tended to overestimate students’ achievement on standardized tests, and correspondingly, underestimated the difficulty of the testing tasks. This suggests teachers may have an overly optimistic view of their students’ performance levels.

Paleczek et al. (2017) examined the variables that influenced teachers’ accuracy when determining second- and third-graders’ reading abilities, such as the students’ decoding and reading comprehension. They found that teachers judged reading comprehension more accurately than decoding at the class level, and that they judged reading comprehension more accurately in the third grade than in the second. Teachers typically did not evaluate students’ reading abilities in the same way as standardized assessments that focus on specific abilities. However, when more skills are considered and the identification process is guided, teachers seem to be able to accurately identify students ARORD (Schmitterer & Brod, 2021).

## Teacher characteristics

Another component that may influence teachers’ decision-making regarding their students’ skills is teachers’ characteristics. To date, few researchers have examined how they may influence decision-making (Südkamp et al., 2012) at different stages of judging students’ performance (Impara & Plake, 1998). The teaching experience is the most frequently examined characteristic (Urhahne & Wijnia, 2021). Ready and Wright (2011) found that new kindergarten teachers with little experience tended to overestimate their students’ literacy skills. Another study, concerning second- and third-graders’ reading abilities (decoding and reading comprehension), found that teachers improved at judging reading comprehension over the years (Paleczek et al., 2017). Another characteristic that has been examined is teachers’ training in the test used in the study; teachers who received training were found to make better decisions regarding their students’ skills than teachers without training (Begeny & Buchanan, 2010).

## The simple view of reading and Arabic literacy

The theoretical framework for this study was grounded in the Simple View of Reading (SVR), first proposed by Gough and Tunmer (1986), where reading comprehension is the product of proficiency in two basic processes and the relationship between them: word decoding and linguistic comprehension (Hoover & Gough, 1990; Kirby & Savage, 2008). To comprehend a text, a reader must not only decode the written words accurately and efficiently but also grasp their linguistic meaning within the context of the ideas being expressed. If either process is impaired or underdeveloped, the reader’s ability to understand the text will be compromised, regardless of their strength in the other component (Catts et al., 2005).

The SVR has been validated across various languages and orthographies with different levels of transparency between spelling and sound (Florit & Cain, 2011; Joshi et al., 2015). Its cross-linguistic robustness underscored the suitability of the SVR for our study focused on Arabic language with unique orthographic and linguistic features that may uniquely influence the relationship between decoding and comprehension (Abu-Rabia & Taha, 2006). By framing our research using the SVR, we aimed to contribute to the growing body of knowledge on how its fundamental processes interact in Arabic readers, potentially uncovering language-specific patterns that could inform targeted instructional strategies and interventions (Asadi & Khateb, 2017).

## Reading fluency and comprehension

There are two main components of effective reading: accuracy and speed (Geva et al., 1997). Reading fluency is based on these at the word level (Meyer & Felton, 1999). Wolf and Katzir-Cohen (2001) presented the developmental definition of reading fluency as referring to the ability to read a word accurately, automatically, and with correct prosody, without struggling with decoding. Along similar lines, Chard and Pikulski (2005) defined reading fluency as the ability to read text accurately, quickly, and with appropriate intonation, with sufficient word-level comprehension to construct the meaning of the text (Chard & Pikulski, 2005). Learning to read requires phonological, orthographic, morphological, semantic, and syntactic processes at the word and text levels (Wolf & Katzir-Cohen, 2001). In addition to learning the alphabetic system, reading also requires decoding, vocabulary, and text comprehension (Becker et al., 2010).

Snow (2002) defined reading comprehension as “the process of simultaneously extracting and constructing meaning through interaction and involvement with written language” (p. 11), which has been noted to be multidimensional (Lipka & Siegel, 2012). Reading comprehension is considered the most complex human skill that involves a cognitive activity (Kendeou et al., 2016). Many studies examining English speakers have shown a relationship between reading fluency and comprehension (e.g., Fuchs et al., 2001; Kim et al., 2012), where the former contributes to the latter (Wolf & Katzir-Cohen, 2001).

## Poor readers and poor comprehenders

Poor readers struggle with word recognition and reading comprehension (Torppa et al., 2007), and the SVR model predicts a negative correlation between decoding and listening comprehension skills in these individuals. This prediction has been supported by studies on young children, and for older poor readers, the model suggests an even stronger negative correlation between these skills. Torppa et al. (2007) found that poor readers in several early-years language and literacy tasks, particularly in phonological awareness, letter knowledge, rapid naming, and vocabulary. Interestingly, certain case studies have followed individuals with poor decoding skills but relatively good reading comprehension, termed “resilient readers” by Jackson and Doellinger (2002). This phenomenon suggests that some poor readers may develop compensatory strategies that allow them to overcome their decoding difficulties, as noted by Hulme and Snowling (1992).

A significant subset of children with reading difficulties constitutes poor comprehenders, defined as readers who exhibit average word recognition skills but lag behind in reading comprehension (Torppa et al., 2007). Notably, these children demonstrate compromised vocabulary skills from an early age and show somewhat below-average performances in various tasks, possibly due to difficulties in understanding the task demands (Nation, 1999). Furthermore, poor comprehenders engage less in reading for fun and have fewer shared reading experiences with their parents before reaching school age compared to good readers (Torppa et al., 2007). While some poor comprehenders may improve over time, those who continue to struggle in later grades may exhibit a clear pattern of deficient oral language and memory skills (Torppa et al., 2007). Nonetheless, their difficulties can easily go unnoticed, as identifying them can present a significant challenge for educators (Leach et al., 2003). This underscores the need for targeted interventions and increased awareness of this specific reading profile in educational settings.

### The present study

This pilot study had three main research arms. First, we investigated which factors Arabic-speaking teachers use to decide on third-grade students’ word reading fluency and reading comprehension. Second, we explored whether Arabic-speaking teachers can accurately identify students who need interventions based on their performance in word reading fluency and comprehension. Third, we looked for characteristics between Arabic-speaking teachers who accurately assess versus those who inaccurately assess the literacy skills of students at risk of reading difficulties ARORD. The significance of this pilot study lay in examining teachers’ decision-making processes and the accuracy of their decisions, serving two critical purposes: developing targeted professional training programs based on teachers’ needs, and creating standardized tests that enable teachers to identify students showing early signs of literacy difficulties. The focus was on native Arabic-speaking teachers in the third grade, as this is a critical period in reading development. While third-grade students have typically developed their reading fluency with vowels and gained basic Arabic reading comprehension skills, they are transitioning from shallow to deep Arabic orthography, a process complicated by reduced vowelization, which makes reading more challenging.

Several studies have sought to determine teachers’ accuracy in deciding student characteristics such as academic achievement, which has become important considering the many national and international reports of students’ low reading comprehension in the upper grades and specifically gaps in reading comprehension among Arabic-speaking students, as shown by the results of the recent the PIRLS study and Meitzav national tests. Elsewhere, Schmitterer and Brod (2021) comprehensively assessed various reading and language skills among third-grade German-speaking students using standardized, normed tests of word reading, sentence reading, text reading, spelling, phonological awareness, and vocabulary size, which allowed for a detailed examination of multiple aspects of reading and language proficiency in the German-language context.

Our pilot study took a more focused approach tailored to the unique characteristics of Arabic language acquisition. We studied two fundamental skills in the developmental stage of Arabic language acquisition: reading at the word level and reading comprehension. This targeted approach addressed the specific challenges presented by the Arabic writing system. Teachers are heavily invested in developing these two skills in their daily lessons, dedicating significant instruction time and resources to strengthening students’ word reading and reading comprehension abilities, which are at the core of their teaching priorities and curriculum implementation. Moreover, we consider that students possess diverse abilities, resulting in different learner profiles, and that different sources of information may be required for each profile to provide an accurate and nuanced assessment. This approach allowed us to capture the heterogeneity of Arabic language learners and tailor our analysis to their individual needs and challenges. By considering these varied profiles and utilizing appropriate assessment methods for each, we aimed to gain a comprehensive understanding of Arabic literacy development, to inform the development of more effective, personalized instructional strategies.

### Methods

#### Participants

This pilot study was conducted as part of a larger longitudinal project that initially followed over one thousand children from kindergarten to elementary school in Israel. In the Israeli education system, the transition from kindergarten to elementary school is characterized by a widespread phenomenon of student dispersion, where children from the same kindergarten may transition to different elementary schools. This phenomenon stems from various considerations, such as flexible registration zones, parental preferences, different educational approaches of schools, and geographical distance. Due to this dispersion and the fact that some schools or families chose not to continue their participation in the longitudinal study, the original sample of one thousand children decreased significantly. Furthermore, the outbreak of COVID-19 led to the suspension of the research in second grade (2020) and frequent lockdowns during third grade (2021), which complicated continued data collection and led to a further reduction in the number of participants.

By third grade, a total of 558 students remained in the study. For this preliminary investigation, we focused on an initial sub-sample of third-grade teachers and students in Arabic-speaking schools in northern Israel, comprising 58 teachers and 112 students from 37 elementary schools that managed and agreed to continue participating in the research despite these challenges. It is important to note that this study did not include entire classrooms; rather, an average of five students participated in each class. The distribution of students per teacher was as follows: 44% of the teachers assessed one student, 11% assessed two students, and 45% assessed three students. Teachers agreed to complete assessments for a maximum of three students. To avoid bias and selection based on teachers’ prior knowledge of students’ abilities, the research team randomly selected up to three students from the five available participants per class. Some teachers, due to heavy workload, opted to assess only one or two students, while others agreed to assess three.

##### Teachers

The preliminary sample included 58 Arabic language teachers whose mother tongue is Arabic. The teachers in this pilot study were not randomly selected but were part of a subset from the larger longitudinal study. All third-grade teachers in the participating Arabic-speaking schools in northern Israel with students from the original cohort were invited to participate in this phase of the research. The teachers included were those who consented to take part. The teachers were predominantly female (N male = 5), and their mean age was 43.44 years (SD = 8.12). All were third-grade teachers; 58 had a bachelor's degree and teaching certification, while 34 teachers had a master's degree. The years of teaching experience ranged from 4 to 36 years (M = 19.93, SD = 5.35). Regarding teachers'religion, the pilot study included 59.4% Muslim, 22% Druze, and 18.6% Christian teachers.

##### Students

This pilot phase included 112 native Arabic-speaking third-graders. All participants attended Arab sector schools in Israel where Arabic is the primary language of instruction. In these schools, students primarily learn to read in Arabic, with Hebrew introduced as a second language and English as a foreign language. All students were from Haifa and the Northern District in Israel. The participants comprised 54 boys and 58 girls, and their mean age was 8.66 years (*SD* = 0.42). The pilot study included 58% Muslim, 33% Druze, and 9% Christian students. We determined their socioeconomics using Israeli Ministry of Education data for each school location, which indicated that overall, participants had a low or medium–low socioeconomic background. Background information on the teachers and students in this preliminary study is presented in Table 1.

**Table 1:** Demographics of the teachers and students

Characteristics	Teachers		Students	
	*N*	%	*N*	%
Gender				
Female	53	91.4	58	51
Male	5	8.6	54	49
District				
North	51	87.9	104	92.9
Haifa	7	12.1	8	7.1
Religion				
Muslim	34	59.4	65	58
Christian	11	18.6	10	9
Druze	13	22	37	33
Dialect				
Villager	21	36.2	30	26.8
Urban	16	27.6	35	31.3
Bedouin	8	13.8	10	8.9
Druze	13	22.4	37	33
	***M***	***SD***	***M***	***SD***
Age in years	43.44	8.12	8.66	0.42
Years of teaching experience	19.93	5.35	-	-
Years of education	17.95	8.75	-	-

## Measures

### Teacher questionnaires

Teachers completed an online questionnaire (a demographic questionnaire, ratings of reading comprehension and fluency abilities, and teachers'source of decision-making). The tools were adjusted to the current pilot study and the Israeli education system and were translated from Hebrew or English to Arabic and adapted for the purposes of this pilot study and the Israeli education system by the Safra language team (created by Lipka, 2022).

## Teachers’ demographic questionnaire

The questionnaire first collects general information about teachers, including their gender, age, education level, student-facing teaching hours, and teaching seniority (developed by Lipka, 2022). In Israel’s elementary education system, a teacher’s role is divided into student-facing teaching hours and preparation hours**.** Student-facing hours include frontal teaching (classroom instruction), individualized instruction (one-on-one or small group sessions), and supervising in-class activities. Preparation hours**,** on the other hand, cover tasks such as lesson planning, grading, creating teaching materials, and participating in meetings and training. In the current pilot study, we measured the time teachers spent actively instructing in the classroom based on the former, which includes time spent lecturing, leading class discussions, and supervising in-class activities.

## Teachers’ rating of reading abilities questionnaire

Here, teachers rated individual students’ reading abilities compared to their peers. The questionnaire is based on a scale from a German study of teachers’ ability to identify third-grade students with reading difficulties (Schmitterer & Brod, 2021), later adapted to Arabic (Abbas-Khatib & Lipka, 2022). It focuses on two main skills: reading fluency and reading comprehension. Teachers rated each student on a scale of one to three (1 “Needs intervention,” 2 “May need intervention,” 3 “Does not need intervention”) for these skills: “Please indicate whether the student needs a reading fluency intervention or not, according to the items on a scale of 1–3.” The reading comprehension component was developed separately by Abbas-Khatib and Lipka (2022). Teachers were asked the following: “Please indicate whether the student needs a reading comprehension intervention or not, according to the items on a scale of 1–3.” The questionnaire demonstrated high reliability, with α = 0.90.

## Teachers’ decision-making questionnaire

This assessed the extent to which teachers relied on different information sources when deciding their students’ reading fluency and reading comprehension (Schmitterer & Brod, 2021, adapted to Arabic by Abbas-Khatib & Lipka, 2022). The questionnaire includes six sources: students’ oral reading abilities, linguistic skills, motivation, and self-perception, parents’ reports, test scores, and vocabulary size. Teachers rated each on a five-point Likert-type scale ranging from one (strongly disagree) to five (strongly agree). An example item is as follows: “Please indicate to what extent you rely on the following information to determine a learner’s (X) reading fluency ability on a scale of one (strongly disagree) to five (strongly agree).” The reliability of the questionnaire was α = 0.72 for the reading fluency section and α = 0.79 for the reading comprehension section. Abbas-Khatib and Lipka (2022) developed a similar questionnaire specifically for research-based sources of information on a student’s reading comprehension skills.

## Students’ tasks

The participants completed several tasks (on oral word reading fluency and reading comprehension) in individual and group sessions at their schools.

- TOWRE Test of Word Reading Fluency (Katzir et al., 2012, is based on Wagner et al. 1999 and adapted to Arabic by Jabour-Danial et al., 2021; Schiff et al., 2006). This test assesses students’ word reading fluency. Participants were asked to read aloud 80 single words as quickly and as accurately as possible in 45 s. The words were ordered with increasing difficulty, and scores were calculated according to the number of correct words provided. The reliability of this test is α = 0.89 (see Table 2).
- TAMAR Test of Reading Comprehension (adapted to Arabic from the TAMAR test in Hebrew by Katzir & Sabag-Shushan, 2018). This test comprises two short texts for each level from third to sixth grade (Shalhoub-Awwad & Hamza, 2021). After reading each text quietly, five multiple-choice questions are presented, each with four options for their answers. Participants mark the correct answers on the computer in a maximum time of 10 min. The score is the sum of the correct answers. The reliability of this task is α = 0.53 (see Table 2).

**Table 2:** Descriptive statistics and reliability for students’ literacy skills tasks

Task	*N*	*M*	*SD*	Min	Max	Reliability
Word reading fluency	112	17.99	8.74	1	34	0.89
Reading comprehension	112	4	2.26	0	10	0.53

## Procedure

This pilot study proceeded after we received approval from the Chief Scientist of the Israeli Ministry of Education and the ethical committee of the Faculty of Education at the University of Haifa, Israel. After parents and teachers granted their permission for study participation (completed consent forms), we randomly selected one to three students from each teacher’s classroom. The students performed their tasks individually in a quiet space at school, led by a research assistant; there were two task sessions, each lasting 45 min. Next, we sent teachers e-links to their three questionnaires, and data were collected with their return.

## Statistical analyses

Statistical analyses were conducted according to the arms of this pilot study. The first examined which data or information teachers use to make decisions about students’ literacy abilities, for which descriptive statistics were calculated using SPSS software. The second concerned teachers’ ability to identify students ARORD in alignment with their actual performance, for which we calculated descriptive statistics in order to identify students who needed an intervention, based on the sample means for the word reading fluency task (M = 17.99, SD = 8.74) and the TAMAR reading comprehension test (M = 4, SD = 2.26). We defined students as ARORD if their raw scores were one standard deviation below the mean performance for the sample, rather than age- or grade-based norms, based on Torppa et al. (2007). In accordance with the Simple View of Reading (SVR) model, we assumed that skill levels in word reading fluency and reading comprehension do not always match. Our analysis focused specifically on teachers'ability to identify students across the different ARORD profiles: students with low word reading fluency only, students with low reading comprehension only, students with both low word reading fluency and low reading comprehension, and students with typical profiles. Using independent-samples *t*-tests, we compared various teacher characteristics between the accurate and inaccurate assessor groups to identify which factors might contribute to better identification of students who require literacy intervention.

## Results

### Indicators for Arabic-speaking teachers’ decision-making

**Fig. 1:**
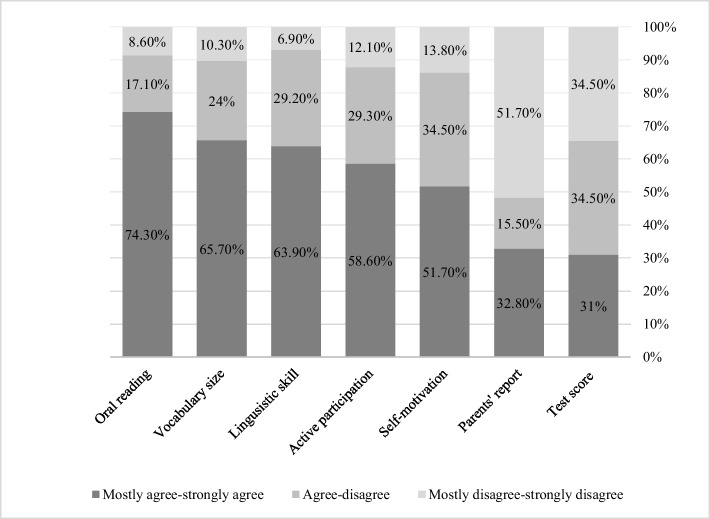
Teachers’ self-reported factors they relied on to reach decisions about third-grade students’ reading fluency

The first research arm examined the factors Arabic-speaking teachers consider when deciding third-grade students’ reading fluency and comprehension. For reading fluency, the results presented in Fig. 1 and Table 3 demonstrate that teachers rely most heavily on factors directly related to students’ literacy skills, chiefly their oral reading (74.30%), vocabulary size (65.70%), and linguistic skills (63.90%). Other frequently used indicators include active participation in class (58.60%) and student motivation (51.70%). Interestingly, teachers reported less reliance on external sources of information, with parents’ reports (32.80%) and test scores (31%) the least frequently used when assessing students’ reading fluency.

**Table 3:** Factors teachers relied on to reach a decision about their third-grade students’ reading fluency

Type of factor	*N*	*M*	*SD*	Mostly agree/strongly agree	Agree/disagree	Mostly disagree/strongly disagree
Oral reading	58	4.18	0.88	74.30%	17.10%	8.60%
Vocabulary size	58	4	1.11	65.70%	24%	10.30%
Linguistic skills	58	3.98	0.99	63.90%	29.20%	6.90%
Active participation	58	3.85	1.07	58.60%	29.30%	12.10%
Self-motivation	58	3.69	0.90	51.70%	34.50%	13.80%
Parents’ reports	58	2.77	1.39	32.80%	15.50%	51.70%
Test scores	58	2.97	1.34	31%	34.50%	34.50%

**Fig. 2:**
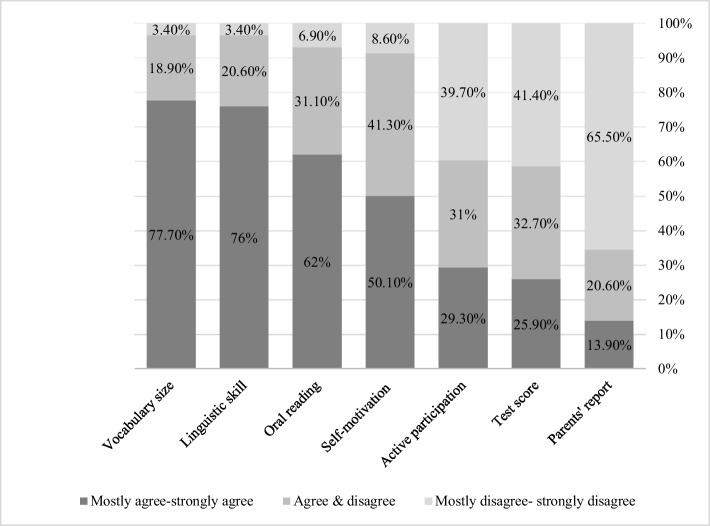
Teachers’ self-reported factors they relied on to reach decisions about students’ reading comprehension

For reading comprehension, as shown in Fig. 2 and Table 4, teachers primarily rely on outcome data such as the vocabulary size (77.70%), linguistic abilities (76%), and oral reading level (62%). Another common factor is their impression of the child’s motivation (50.10%). The least relied upon factors for assessing reading comprehension are active participation (29.30%), test scores (25.90%), and parents’ reports (13.90%).

**Table 4:** Factors teachers relied on to reach decisions about their third-grade students’ reading comprehension

Type of Factor	*N*	*M*	*SD*	Mostly agree/strongly agree	Agree/disagree	Mostly disagree/strongly disagree
Vocabulary size	58	0.20	0.77	77.70%	18.90%	3.40%
Linguistic skills	58	4.20	0.71	76%	20.60%	3.40%
Oral reading	58	3.96	0.96	62%	31.10%	6.90%
Self-motivation	58	3.73	0.98	50.10%	41.30%	8.60%
Active participation	58	2.80	1.24	29.30%	31%	39.70%
Test scores	58	2.70	1.26	25.90%	32.70%	41.40%
Parents’ reports	58	2.26	1.09	13.90%	20.60%	65.50%

## Agreement between Arabic-speaking teachers’ ratings and students’ actual performance

**Fig. 3:**
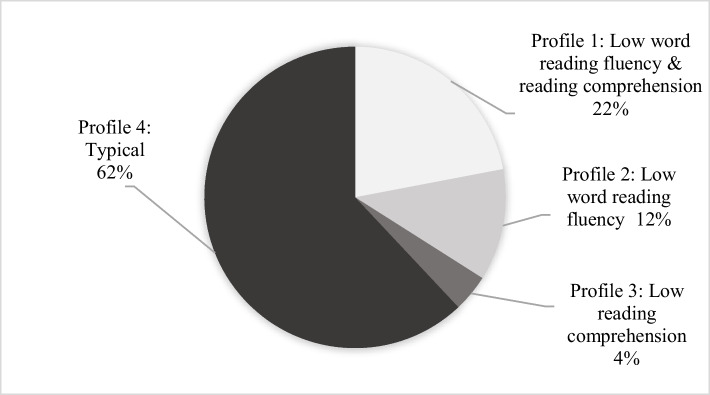
Classifying students according to the four different profiles of word reading fluency and reading comprehension (*n* = 112)

The second research arm examined the accuracy of teachers’ assessments of students’ word reading fluency and comprehension. We employed a two-stage approach, where we first randomly selected students from classes whose teachers agreed to participate in this pilot study. After these students completed two tasks, a word reading fluency task using the TOWRE test and a reading comprehension task using the TAMAR test—we moved on to the second stage. Here, teachers filled out individual questionnaires for each student, selecting “Needs intervention,” or “Does not need intervention” for word reading fluency and reading comprehension skills separately. The quantitative data was then entered into an SPSS database, allowing us to identify students’ ARORD. We identified students at risk using a criterion of one standard deviation below the sample mean performance. Importantly, this identification method was not based on age or grade-based norms. Students were classified as at-risk when they scored below threshold on either word reading fluency (M = 17.99, SD = 8.74) or reading comprehension (M = 4, SD = 2.26). This methodological approach yielded four distinct performance profiles, as van be seen in Fig. 3:

1. Low word reading fluency and reading comprehension: This profile comprised 25 students (22%) who demonstrated difficulties in both Word reading fluency and comprehension, thus facing the greatest struggle with reading tasks.
2. Low word reading fluency: This group included 13 students (12%, *n* = 112) who exhibited difficulties solely in Word reading fluency while maintaining adequate reading comprehension skills. This profile suggests that these students may face specific challenges related to word recognition speed or accuracy.
3. Low reading comprehension: This was the smallest group, consisting of 5 students (4%) who struggled exclusively with reading comprehension tasks but demonstrated adequate Word reading fluency. This profile indicates that these students can read words efficiently but may have trouble extracting or constructing meaning from the text.
4. Typical: This profile included 69 students (62%) who demonstrated solid performance in both word reading fluency and reading comprehension. These students exhibited a typical pattern of reading development, efficiently decoding words while successfully deriving meaning from the text. Unlike the other profiles, these readers did not display significant difficulties in either reading domain. Their performance was one standard deviation above the average. These profiles, illustrated in Fig. 3, provide a nuanced picture of the ways reading difficulties can manifest in students, highlighting the importance of targeted interventions based on individual needs.

**Fig. 4:**
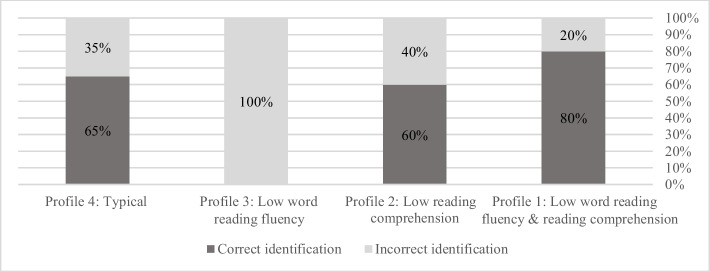
Proportions of students in each profile, as identified by teachers

**Fig. 5:**
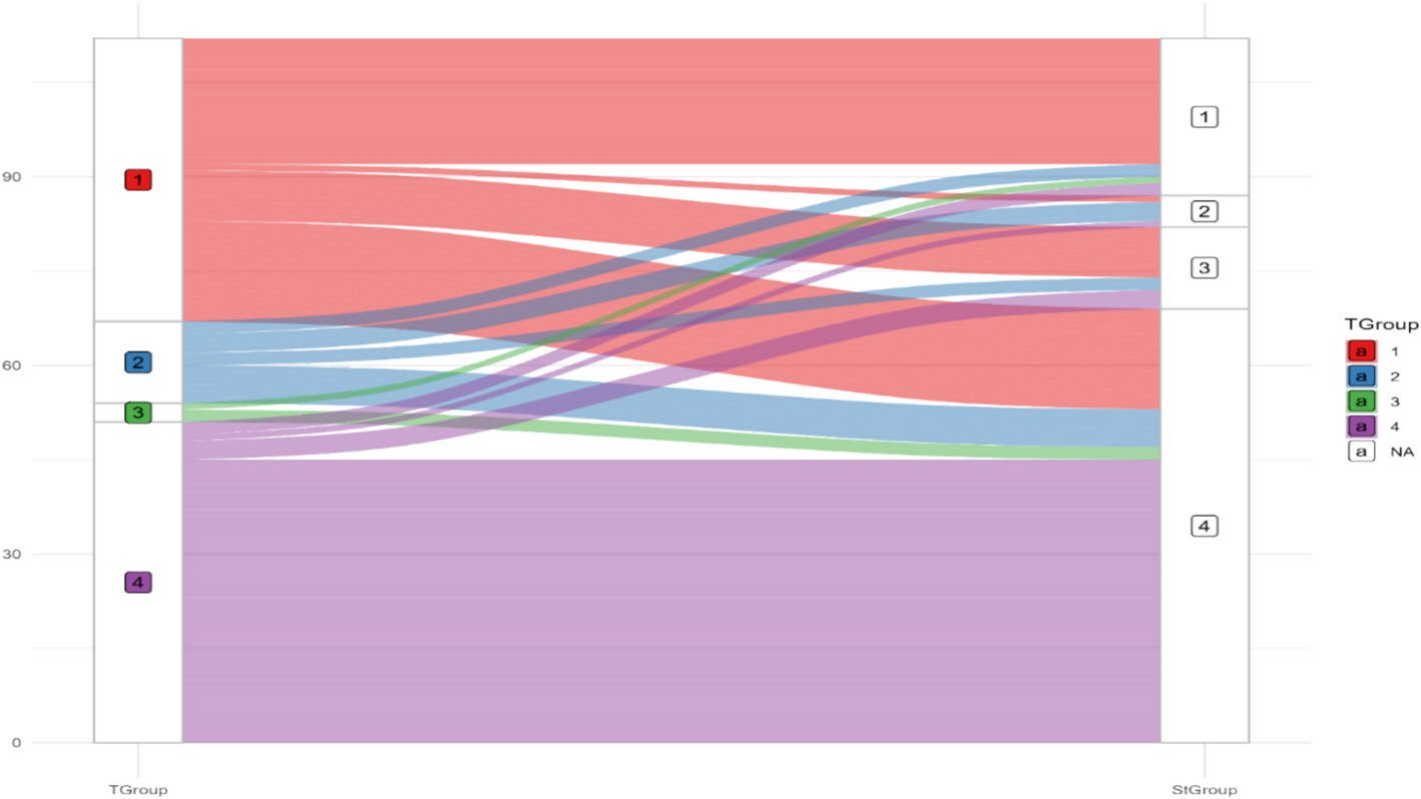
Reading profile identification flow: Comparison between teacher evaluations (TGroup) and actual classifications (StGroup)

**Fig. 6:**
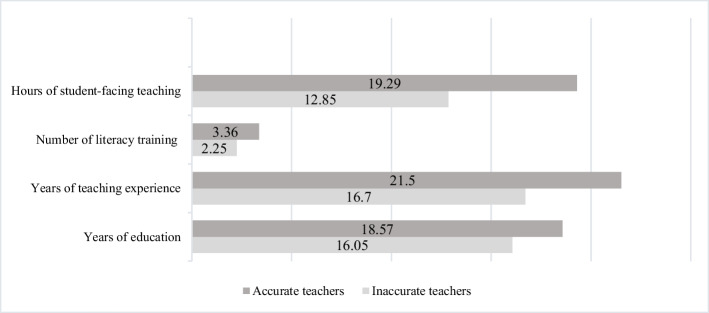
Means of Arabic-speaking teachers’ characteristics in both study groups

Next, we examined teachers’ ability to accurately identify at risk and typical students in their classroom. The results revealed varying accuracy in teacher identification across different profiles (see Fig. 4): For Profile 1 (difficulties in both word reading fluency and comprehension), teachers correctly identified 80% (20) of students and misidentified 20% (5). For Profile 2 (difficulties in reading comprehension only), teachers correctly identified 60% (3) of students and misidentified 40% (2). In contrast, for Profile 3 (difficulties in word reading fluency only), teachers failed to identify any of the students with fluency difficulties (0%). For Profile 4 (students with typical reading abilities, i.e., those who performed at or above the threshold in both domains), teachers demonstrated 65% (45) accuracy in correctly identifying these students, while 35% (24) were misidentified as needing intervention. Overall, the general accuracy rate of teachers in correctly identifying students was 61% (68 out of 112) (Figs. 4, 5, and 6).

Next, as can be seen in Fig. 6, the analysis revealed significant patterns in the accuracy of teacher evaluations compared to students'actual classifications regarding reading difficulties. The Sankey diagram demonstrated that teachers showed highest accuracy when identifying students with broader reading difficulties (Profile 1), correctly classifying 80% of students with combined word reading fluency and comprehension issues. However, they struggled considerably with identifying isolated difficulties, with lower accuracy for students with comprehension-only difficulties (Profile 2), followed by the lowest accuracy observed in students with fluency-only difficulties (Profile 3), where no correct identifications were made. Additionally, teachers tended to over-identify students as requiring intervention, particularly those with typical reading abilities (Profile 4).

The final research arm examined differences between Arabic-speaking teachers who accurately assessed literacy skills and those who did so inaccurately for students at risk of reading difficulties (ARORD). The study hypothesized that these two groups of teachers would differ in certain characteristics. To test this hypothesis, an independent samples t-test was conducted. Table 5 and Fig. 6 presents the comparison data for the study groups. The findings revealed a statistically significant difference in student-facing teaching hours [t(32) = 1.74, *p* < 0.05]. Teachers who accurately identified students reported more student-facing teaching hours (M = 19.29, SD = 11.54) compared to those who inaccurately identified students (M = 12.85, SD = 9.99). However, contrary to the research hypothesis, no significant differences were found between the groups in other measures: years of education [t(32) = 1.35, *p* = 0.093]; Years of teaching experience [t(32) = 1.48, *p* = 0.074]; number of literacy training [t(32) = 0.58, *p* = = 0.567].

**Table 5:** Means and standard deviations of Arabic-speaking teachers’ characteristics in both study groups

		Accurate teachers			Inaccurate teachers		*T*	Cohen’s *d*
	*N*	*M*	SD	*N*	*M*	SD		
Hours of student-facing teaching	14	19.29	11.54	20	12.85	9.99	1.74*	0.60
Number of literacy training	14	3.36	7.74	20	2.25	3.14	0.58	0.20
Years of teaching experience	14	21.5	10.36	20	16.70	8.49	1.48	0.52
Years of education	14	18.57	5.96	20	16.05	4.88	1.35	0.47

*p < 0.05 (with “Accurate Teachers” group and “Inaccurate Teachers” group)

## Discussion and conclusion

Recent national and international assessments have revealed a consistently low reading achievement among Arabic-speaking students in Israel’s education system, highlighting a significant literacy challenge that requires immediate attention. In light of these concerning statistics, understanding teachers’ decision-making processes is crucial to improve literacy outcomes, particularly at the critical third-grade juncture. This grade level represents a pivotal transition point where Arabic-speaking students move from reading voweled to unvoweled text, requiring them to develop new reading strategies while simultaneously using reading as a tool for learning across subjects. Given that teacher judgment plays a vital role in identifying students at risk of reading difficulties and determining appropriate interventions at this crucial stage, this pilot study examined three key aspects of Arabic-speaking teachers’ decision-making processes: their primary sources of information when assessing reading skills, their accuracy in identifying students needing an intervention, and the characteristics that distinguish accurate from inaccurate teachers. Our findings revealed several important patterns: teachers primarily relied on observational data rather than standardized assessments, showed varying levels of accuracy in identifying different profiles of reading difficulties, and demonstrated a better assessment accuracy when they had more student-facing teaching hours.

Regarding their information sources, we found that teachers in our study relied more on observational data than on standardized measures when assessing both reading fluency and comprehension. For reading fluency, they primarily relied on oral reading performance (74.30%), vocabulary knowledge (65.70%), and linguistic abilities (63.90%), while for reading comprehension, they emphasized vocabulary size (77.70%), linguistic skills (76%), and oral reading level (62%). In contrast, teachers placed minimal emphasis on standardized test scores (25.90%–31%) and parent reports (13.90%–32.80%). This pattern contrasts with Schmitterer and Brod (2021)**,** who found that teachers primarily relied on cognitive outcome data when available, whereas in our pilot study, teachers exhibited a stronger preference for directly observable indicators like oral reading and linguistic skills. While this reliance on direct observation aligns with research by Perfetti and Stafura (2014) on core reading competencies and Rasinski et al. (2011) on reading fluency components, the limited use of objective, standardized measures raise concerns about the comprehensiveness of teachers’ assessments and their ability to detect subtle reading deficits.

When examining our second research focus, concerning teachers'accuracy in identifying students at risk of reading difficulties (ARORD), we found varying levels of success across different reading profiles. Building on Torppa et al.’s (2007) work and using the Simple View of Reading (SVR) model as our framework, we identified four distinct profiles. Teachers demonstrated the greatest accuracy (80%) in identifying students with both word reading fluency and reading comprehension difficulties (Profile 1). For students struggling solely with reading comprehension (Profile 3), teachers correctly identified 60% of these students. However, the most striking finding pertains to students with isolated word reading fluency difficulties (Profile 2). Our analysis revealed that teachers failed to identify any students within this category (0% accurate). For students with typical reading abilities (Profile 4), teachers exhibited a 65% accuracy rate. However, 35% were mistakenly classified as needing intervention, indicating potential over-identification issues that could lead to unnecessary resource allocation. These findings highlight the significant challenge teachers face in identifying specific fluency difficulties when they occur without accompanying comprehension problems.

These findings can be interpreted within the framework of the SVR model, which posits that reading comprehension is the product of decoding and linguistic comprehension (Gough & Tunmer, 1986). The teachers'higher accuracy (80%) in identifying students with both word reading fluency and reading comprehension deficits suggests they rely on observable indicators of overall reading failure rather than isolating specific skill deficits. These students likely exhibit clear and multiple signs of reading difficulties that are readily apparent in classroom activities. This supports the SVR model's assertion that both components are essential for successful reading. However, our findings reveal significant challenges in identifying single-skill deficits, with particularly concerning results for fluency difficulties (0% accuracy). These lower accuracy rates challenge the assumption that teachers can effectively disentangle the two components in their assessments. According to the SVR model, students with isolated deficits in either fluency or comprehension should be equally recognizable, yet our results demonstrate that teachers struggle significantly to pinpoint these specific difficulties, especially isolated fluency problems.

To understand this pattern, we must examine the inherent differences in assessing various language skills. Spelling offers clear right or wrong answers and produces tangible written outputs, making it easily assessable through routine tasks. In contrast, reading fluency requires evaluations of speed, accuracy, and rhythm, which are not immediately observable without dedicated assessment. Similarly, reading comprehension involves complex cognitive processes that are challenging to assess in regular classroom activities. While spelling can be evaluated frequently with simple tools, accurately assessing reading fluency and comprehension demands dedicated time, attention, and more sophisticated methods. The lack of explicit fluency assessment tools in teachers’ training may contribute to their inability to recognize isolated fluency deficits.

Our findings align with previous research in this field. Paleczek et al. (2017) found that teachers judged reading comprehension better than reading skills. Our results also echo recent studies by Asadi et al. (2017) and Mansour-Adwan et al. (2023) on Arabic language difficulties, reinforcing the notion that teachers predominantly rely on holistic observations of reading performance rather than component-specific assessments. This approach aligns with Catts et al.’s (2006) emphasis on straightforward identification methods in classroom settings. A meta-analysis by Südkamp et al. (2012) further highlighted the variability in teacher judgment accuracy across domains and assessment types. The concerningly low accuracy in teacher identification, particularly for isolated fluency deficits, can be attributed to several interconnected factors. In the classroom setting, reading is an integrated activity where fluency and comprehension naturally intertwine, making it challenging to isolate individual components. Teachers typically observe students engaging with whole texts, responding to content, and demonstrating overall reading proficiency, which provides a holistic but potentially incomplete view of their specific abilities. As Begeny and Buchanan (2010) argued, insufficient teacher training and practice in conducting targeted assessments contribute to difficulties in accurately judging students'specific reading skill levels.

This discrepancy highlights a potential gap in teacher preparation regarding the distinct identification of reading fluency versus reading comprehension difficulties. Our study particularly demonstrates that Arabic-speaking teachers may be substantially better at identifying children with combined reading difficulties rather than isolated skill deficits, suggesting a critical need for targeted professional development in component-specific assessment techniques.

For our third research focused on teacher characteristics, we found that student-facing teaching hours emerged as a crucial factor in assessment accuracy. Teachers who spent more time in direct instruction demonstrated superior ability to identify students with reading difficulties. This finding aligns with Blatchford et al. (2011), who demonstrated that more active interaction with students enhances instruction quality. Similarly, Paleczek et al. (2017) suggest that such direct engagement might improve the accuracy of teachers’ diagnostic decisions. From an SVR perspective, increased direct interaction with students appears to enhance teachers'understanding of how decoding and comprehension components contribute to reading difficulties. Greater exposure to students'reading behaviors in both reading fluency and reading comprehension tasks enables teachers to develop a more nuanced understanding of these challenges, leading to more informed assessments. Teachers with more student-facing hours likely develop enhanced abilities to observe, understand, and monitor their students'academic difficulties effectively. Notably, contrary to our initial hypotheses, other factors such as the number of literacy training sessions, Years of teaching experience, and years of education showed no significant differences between accurate and inaccurate decision-makers. This unexpected finding suggests that practical classroom experience may be more valuable than formal education in developing assessment accuracy.

In conclusion, the critical role of third-grade teachers in identifying students'reading fluency and comprehension performance emphasizes the importance of our findings. As highlighted by Torgesen (2002), early identification is crucial for preventing reading difficulties. Our results indicate a need for improved teacher training specifically focused on identifying diverse profiles of at-risk students, especially in cases where students excel in one area but struggle in another. It is important to emphasize that misidentifying typical students and incorrectly classifying them as struggling learners can lead to a waste of valuable resources in the education system. This misidentification may result in directing support and intervention resources to students who don't actually need them, while students who truly need assistance may receive less attention. Moreover, incorrect classification can negatively impact students'self-image, generate low expectations from teachers, and adversely affect their motivation and academic performance over time. Research shows that educational labels can create a negative “Pygmalion effect,” where teacher expectations influence student development (Rosenthal & Jacobson, 1968).

In accordance with Moats’ (2009) recommendations, comprehensive training should emphasize explicit instruction in distinguishing between decoding difficulties and comprehension difficulties within the SVR model and include training in accurately identifying students who truly need intervention versus those whose performance falls within the typical range. Additionally, developing standardized assessment tools adapted to the Arabic-speaking educational context is essential for improving teachers'decision-making processes and reducing misidentification rates in both directions. By improving the accuracy of teacher assessments and providing them with the necessary tools to correctly distinguish between reading fluency difficulties, reading comprehension difficulties, and typical performance, we can allocate resources more efficiently, better support students at genuine risk, and improve literacy outcomes in Arabic-speaking classrooms.

## Limitations of this study

This pilot study serves as a preliminary review of the topic and includes several limitations that should be considered. The primary limitation is the small student sample size. While data were collected from 37 elementary schools, providing a diverse range of educational contexts, each teacher assessed the literacy skills of only one to three students in their classroom. This sampling approach allows for in-depth, context-rich observations but results in a relatively small overall sample size. Another limitation concerns the measurement properties of the TAMAR reading comprehension test (α = 0.53). This low reliability is partly due to the test being in its developmental stages and covering a broad grade range (third to sixth grade), but it also reflects a broader challenge in the field regarding the lack of standardized Arabic reading comprehension assessments in the Israeli educational context. Consequently, the statistical power of this study is limited, preventing the use of more complex analytical techniques such as multilevel modeling, despite the hierarchical nature of the data (students nested within classrooms and schools).

Since this is a pilot study, expanding the sample size in future research is crucial, ideally including multiple students from each classroom. A larger sample would not only improve the precision of statistical estimates but also enable the application of more advanced analytical methods, which could reveal more nuanced patterns and relationships within the data. Another significant limitation pertains to the sources of information included in this pilot study to understand the data or the factors that Arabic-speaking teachers rely on when assessing the literacy skills of their third-grade students. While this study provides important preliminary insights, it may not fully capture the range of resources and information channels available to teachers. Future research could expand on this by incorporating additional sources, such as standardized test scores, parent–teacher conferences, student portfolios, or peer assessments.

### Implications and recommendations

The findings of this pilot study have significant implications for educational practice, policy, and research in the context of Arabic literacy instruction in Israel. Given the concerningly high rates of reading difficulties among Arabic-speaking students and teachers’ critical role in early identification, our recommendations focus on enhancing their assessment accuracy, improving instructional practices, and developing more effective support systems for both teachers and students. These recommendations are particularly addressed at managing the transition challenges third-grade students face as they move from voweled to unvoweled text, when accurately identifying reading difficulties is crucial to instigating targeted interventions.

At the classroom level, teachers should adopt a more balanced use of observations and standardized assessments, as our results show they are over-reliant on subjective measures. We recommend that teachers should regularly use objective assessment tools such as the TOWRE and TAMAR tests alongside classroom observations, while systematically documenting students’ reading progress in both fluency and comprehension. Moreover, given our finding that increased student-facing teaching hours correlate with a better assessment accuracy, we propose that implementing regular one-to-one reading sessions with students will enhance teachers’ ability to identify and address specific reading challenges. These sessions will provide a structured framework for dynamic and ongoing assessment of students'reading abilities. In the Israeli education system, where classrooms often have around 30 students, teachers face significant challenges in conducting in-depth individual assessments during regular lessons. Currently, there is no standardized system for reading assessment in elementary schools, leaving teachers to rely on general impressions formed during instruction. Through these one-to-one sessions, teachers will be able to assess students’ oral reading fluency, evaluating accuracy, speed, and prosody—while also gauging comprehension through discussions about the text. This focused approach will allow educators to pinpoint specific difficulties, such as decoding struggles or fluency deficits, and adapting their instruction to meet each student's needs.

Schools can play a crucial role in implementing these recommendations through a well-planned, phased approach. Instead of simply increasing student-facing teaching hours, the focus should be on optimizing them by reducing the administrative workload and utilizing additional support staff. This approach ensures higher quality and more focused instruction. Additionally, regular assessment periods should be seamlessly integrated into daily learning routines through brief formative assessments (5–10 min) within lessons, rather than allocating separate time slots for testing, while leveraging digital tools to enhance efficiency and accuracy.

Support from educational decision-makers must include significant structural changes. Beyond considerations related to class sizes, resources should be allocated to establish dedicated time slots for short (15–20 min) individual tutoring sessions, which differ from regular instruction by focusing on specific skills identified through diagnostic assessments. To ensure effective implementation, these sessions can be conducted by teaching assistants or reading specialists. Moreover, collaborative assessment teams should be established, allowing teachers to cross-check data and refine instructional insights, accompanied by professional development tailored to each stage of implementation. Finally, clear protocols must be developed for identifying and supporting students with different reading difficulty profiles as part of a comprehensive, system-wide approach that ensures gradual and sustainable improvements in school structures.

Without comprehensive systemic support and meaningful structural change, any attempt to implement these recommendations will be superficial and unlikely to yield the desired outcomes. Achieving significant progress requires smart investment, strategic reorganization, and meticulous planning to ensure that every student receives tailored support and that the entire system can foster long-term, sustainable improvements in teaching and learning quality.

At the policy level, there is a pressing need to invest in developing standardized Arabic reading assessment tools suitable for the Israeli context. Policymakers should create comprehensive guidelines for the early identification of reading difficulties that account for Arabic’s unique linguistic features, while also allocating resources for professional development programs focused on reading assessment. Additionally, crucial steps forward are establishing standardized protocols for transitioning students from voweled to unvoweled text and developing policies that support increased student-facing teaching hours in Arabic language instruction.

Furthermore, teacher training programs should be enhanced to better prepare educators for the challenges of teaching all students to read. The enhancements should provide teachers with practical experience in using both observational and standardized assessment tools, specific training on Arabic language features that impact reading acquisition, and workshops focused on the transition from voweled to unvoweled text. Moreover, incorporating training on data-driven decision-making in reading assessment will strengthen teachers’ ability to identify different profiles of reading difficulties accurately.

The implementation of these recommendations could address several critical issues identified in our pilot study for Arabic-speaking students in Israel, supporting a better identification of students who are struggling and more targeted and effective reading interventions for these students.

### Future research directions

This pilot study’s findings illuminate several essential pathways for future research on Arabic literacy assessment and intervention. We recommend that future research should employ larger sample sizes, with multiple students per classroom, enabling more sophisticated statistical analyses, such as multilevel modeling to better understand classroom-level effects. Furthermore, it is crucial that researchers investigate interventions that are effective for the different risk profiles we identified (combined difficulties, fluency only, and comprehension only), particularly given Arabic’s unique linguistic features and the voweled to unvoweled transition in third grade.

The development and standardization of Arabic reading assessments for the Israeli educational context emerge as further critical needs. Our findings highlight the current limitations in assessment tools, especially for reading comprehension. Future work should focus on creating reliable, culturally appropriate instruments that account for Arabic’s diglossic nature and complex orthography. Additionally, longitudinal studies examining how teacher assessment accuracy evolves over time could provide valuable insights into expertise development, particularly during critical transition periods. Finally, research evaluating the impact of professional development on teachers’ identification accuracy could inform the development of more effective training programs, ultimately improving the support for struggling readers.

## Data Availability

The datasets analyzed during the current study is not publicly available due to ongoing related research projects by collaborating research group.
